# Predator-prey dynamics stabilised by nonlinearity explain oscillations in dust-forming plasmas

**DOI:** 10.1038/srep24040

**Published:** 2016-04-05

**Authors:** A. E. Ross, D. R. McKenzie

**Affiliations:** 1 School of Physics, A28, University of Sydney, Sydney, NSW 2006, Australia

## Abstract

Dust-forming plasmas are ionised gases that generate particles from a precursor. In nature, dust-forming plasmas are found in flames, the interstellar medium and comet tails. In the laboratory, they are valuable in generating nanoparticles for medicine and electronics. Dust-forming plasmas exhibit a bizarre, even puzzling behaviour in which they oscillate with timescales of seconds to minutes. Here we show how the problem of understanding these oscillations may be cast as a predator-prey problem, with electrons as prey and particles as predators. The addition of a nonlinear loss term to the classic Lotka-Volterra equations used for describing the predator-prey problem in ecology not only stabilises the oscillations in the solutions for the populations of electrons and particles in the plasma but also explains the behaviour in more detail. The model explains the relative phase difference of the two populations, the way in which the frequency of the oscillations varies with the concentration of the precursor gas, and the oscillations of the light emission, determined by the populations of both species. Our results demonstrate the value of adopting an approach to a complex physical science problem that has been found successful in ecology, where complexity is always present.

Dusty plasmas are common throughout the universe, occurring around many types of objects from quasars to comets. Dust formation by plasmas is one of the mechanisms proposed for the creation of interstellar dust^1^. When comets enter stellar radiation fields, plasmas are created that form particulates^2^. In everyday life, candle flames containing soot particulates are a common example of a dust-forming plasma^3^,^4^,^5^,^6^. In the laboratory, dust-forming plasmas are being studied as sources of nanoparticles with many uses in medicine, electronics, and solar cells^7^,^8^,^9^,^10^. Dust is created in plasmas when they contain a precursor gas that is broken down into reactive fragments by collision with electrons in the plasma. Laboratory plasmas are sustained by external electrical input, either radio frequency (rf) or direct current (dc). The reactive fragments form into dust particles that grow in size and are eventually lost from the plasma^8^,^11^,^12^,^13^,^14^,^15^,^16^,^17^,^18^. Nanodots are a type of nanoparticle made in this way, with potential uses as biosensors in medical diagnostics^10^.

In the laboratory, it is striking that the light emission from a dust-forming plasma oscillates over timescales of the order of tens of seconds, often with diminishing amplitude^19^,^20^,^21^,^22^,^23^. Other properties exhibit similar oscillatory behaviour as well, such as electron density^19^,^24^, IR absorbance^20^,^24^, dust particle mean charge^25^, ion current^25^, laser scattering and transmission^19^,^22^, and mass spectrometry^12^,^22^. These oscillations are the signature of the cyclic phenomena of dust particle nucleation, growth, and loss that have been related to particle size distribution in dust-forming plasmas^23^,^26^. Competing theories have been proposed to explain the detailed physics and chemistry of particle nucleation and cluster growth^8^,^11^,^12^,^13^,^14^,^15^,^16^,^17^,^18^ while problems remain in understanding some basic features of the behaviour. A deeper understanding of whether and how the oscillations can be controlled is a problem of practical significance.

Dust-forming plasmas are complex systems, a feature they share with ecosystems. Both have a considerable variety of species present, with interactions between them. In this work, we aim to capture the essential physics of the dusty plasma problem by casting it into a problem of population dynamics, where the plasma (consisting of electrons and ions) and the dust interact, producing time-dependent oscillations in the populations of dust and plasma. We seek to understand observational data related to this phenomenon. While a wide range of measurements have been reported, and some models developed, there are universal observations that are still not understood, including period dependences and a phase difference between components^19^,^26^. Although they oscillate with the same frequency, the electron density peaks first, before the particle volume density peaks^19^,^21^,^22^,^26^. To date, no model has successfully explained this aspect of the behaviour. It is notable that whilst the ion current and dust particle mean charge, and therefore size, oscillate, the electron temperature may rise continuously, not correlating with cyclic behaviour^25^. Therefore, it is appropriate in the interests of simplicity to neglect electron temperature when modelling cyclic behaviour, and concentrate on the oscillatory aspects of two interacting populations.

The predator-prey problem is addressed in the classic works of Lotka^27^, Volterra^28^, and May^29^. Behaviour in which the predator and prey populations oscillate indefinitely is the solution of the original problem as set out by Lotka and Volterra^29^,^30^,^31^,^32^. A foundational study linked oscillations in the populations of the snowshoe hare and its predator the lynx as measured over 200 years in Canada, by noting periodically recurring pronounced variations in the quantities of fur pelts received by the Hudson’s Bay company^33^,^34^. As originally conceived, the Lotka-Volterra solution was expressed as the following pair of coupled linear first-order differential equations^27^,^28^:


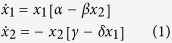


where *x*_*1*_ is the prey population, *x*_*2*_ is the predator population, *α* and *β* are coefficients describing the birth rate and predation rate of the prey, while *γ* and *δ* describe the death rate and birth rate of the predator. The model has oscillating solutions in which both populations increase and decrease periodically and out of step.

Predator-prey modelling has been used to extract understanding from complexity in many contexts: chemical reactions^35^, astronomy^36^, economics^37^, and evolutionary game theory^38^. In climate modelling, the manner in which the structure and behaviour of atmospheric clouds are treated has a significant effect on overall climate sensitivity. This problem has been framed as a predator-prey situation where the rain acts as the predator, the cloud the prey, and the aerosol particles modulate the predator-prey response. Cloud droplets form on aerosol particles and then coalesce to form rain drops, which then consume the cloud and remove its cooling ability. The feedback mechanisms associated with the aerosol-cloud-precipitation system are many and complex, and had proven difficult to incorporate into climate models^39^,^40^. The predator-prey solution enabled the creation of a predictive model, which has proven itself reliable and accurate^41^.

**Our model**. We now describe dust-forming plasmas as a predator-prey system, in which the dust particulates are the predators and the electrons the prey. Figure 1 depicts the processes by which the species interact. The dust depletes the plasma by causing electrons and ions to recombine on its surfaces. The dust is therefore a predator of the plasma. Another process at work is the loss of electrons and ions from the plasma as has previously been studied by us^42^. A key finding using a simple feedback model was that a glow discharge plasma is not stable unless the losses of plasma are dependent on the concentration of electrons to a power greater than one; in other words, the rate of loss of electrons is more than linearly dependent on the electron concentration.

The problem of stabilising wild oscillations is a common theme across disciplines that use predator-prey modelling, and a variety of approaches have been taken to lead to population stability. Nonlinear stability theory has been considered as a means of stabilising predator-prey interactions^37^,^43^, as has the introduction of a firm population limit imposed on one or both of the populations^44^. In keeping with our previous findings, here we include a nonlinear loss term as a natural way of inducing stability.

The model is expressed as coupled equations resembling the original Lotka-Volterra equations by writing the “prey” population as the electron concentration *x*_*1*_ and the “predator” population as the particle material concentration *x*_*2*_. An additional term is added to include the nonlinear loss of the prey. Our resultant equations are as follows:


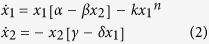


where *n* > 1. The model parameters are listed in Table 1 together with their relation to the dusty plasma environment. Equation set [2] represents an extension of the classic Lotka-Volterra equations, which are recovered when *k* = 0.

Note that the dust population is in our definition a quantity that reflects the concentration of dust material, defined as the mass of dust per unit volume. This is done because the main measure of dust concentration is by light scattering which is greater for larger particles as well as for larger concentrations of particles, preventing a convenient separation into particle number and particle size. The predator loss term γ indicates the loss of grown particles from the plasma, and it incorporates the gravitational force and the ion drag force, both directly proportional to the total mass of dust per unit volume, as in the definition of *x*_*2.*_

The electron and ion loss exponent *n* reflects the order of the reactions that cause plasma loss. One of these reactions is recombination between an electron and an ion, according to the reaction *e*^−^ + *Ar*^+^ → *Ar* where an argon plasma is used as an example. This is a second-order reaction, since its rate depends on the populations of two species: electrons and ions. The population of electrons is approximately equal to the population of ions in normal laboratory plasmas, as a result of the overall charge neutrality, giving a loss rate of electrons proportional to *x*_*1*_^*2*^, so for recombination, *n* is 2. Another process that causes loss is the loss of electrons to the chamber wall, which is a reaction of order 1, since it depends directly on the population of electrons in the chamber. Hence the resultant *n* falls between 1 and 2, since it reflects both loss mechanisms.

The modified Lotka-Volterra equations [2] have a class of solutions in which the populations of prey (electrons in a plasma) and predators (dust particles in a plasma) oscillate out of phase and with diminishing amplitude. This is the same behaviour as reported in measurements of electron and dust populations in dust-forming plasmas^19^,^21^,^22^,^23^.

It is useful to plot the predator population as a function of the prey population, to reveal the basic behaviour of the solutions. For the relevant class of solutions, this plot spirals in to a point of stability, as shown in Fig. 2. In this section we show that solutions of the modified Lotka-Volterra equations give a great deal of insight into the behaviour of dust-forming plasmas. They contain within them an explanation of how the period of the oscillations varies with precursor gas concentration and with electrical power input to the plasma.

The choice of parameters can result in a steady state, a perpetual oscillation, a convergence to equilibrium, or the demise of one or the other, or both, of the species. Analysis of the modified Lotka-Volterra equations [2] allows an assessment of when a discharge might stabilise, if at all. Stability primarily depends on *n* and *k*, because the nonlinear loss is necessary for stability^42^. The modified Lotka-Volterra equations comprise a system of two nonlinear ordinary differential equations. Equilibrium points can be calculated for both the prey population (*x*_*1*_) and the predator population (*x*_*2*_) separately, by setting the opposite population’s derivative to zero. Simple algebra yields:


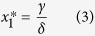


and


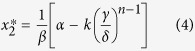


as the equilibrium points. Stability analysis requires linearising the modified Lotka-Volterra equations about the equilibrium points to produce the Jacobian, or community, matrix *J*^45^:


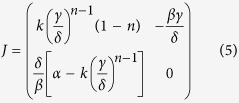


Evaluating 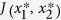 yields the eigenvalues


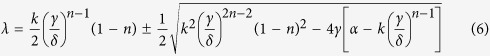


Figure 3 shows eigenvalues for the solution to the modified Lotka-Volterra equations calculated for α = β = 1.5, and γ = δ = 4, as a function of *n* and *k*. This set of parameters allows a wide range of eigenvalues to be seen in a relatively small *n*-*k* parameter space. For much of the *n*-*k* parameter space shown, the eigenvalues are complex. However, where *n* = 1, the eigenvalues are purely imaginary, and where *n* and *k* are large, they are purely real.

The eigenvalues of the Jacobian indicate the stability behaviour of the system^45^. When the eigenvalues are purely imaginary, the dynamics of the system are on the cusp between stability and instability. This case is known as *neutral stability*, in which the system oscillates around equilibrium, without moving closer or farther away. The standard Lotka-Volterra equations show this kind of stability, which gives rise to perpetual oscillations of constant amplitude. Eigenvalues that are complex indicate oscillatory systems, where the predator-prey population plot is spiral in shape. Stability is determined by the sign of the real part of the complex eigenvalue: if it is negative, the system spirals in to an equilibrium point; if it is positive, the system spirals away from the equilibrium point. Population oscillations in time are damped for the stable spiral, while they increase without bound for the unstable spiral. Purely real eigenvalues that are negative indicate the presence of a *stable node*, where the system quickly equilibrates to a stable point without oscillating. Purely real positive eigenvalues yield an unstable node, where the system neither oscillates nor equilibrates.

Figure 4 shows the stability properties of the system corresponding to the eigenvalues shown in Fig. 3. Much of the *n*-*k* parameter space shows stable behaviour, with stable spirals yielding to stable node behaviour where *n* and *k* are large. Small *n* and *k* produce unstable spiralling, and the special case of neutral stability is seen along most of the line where *n* = 1. It is interesting to note that the line of neutral stability does not go all the way to *k* = 0; rather, it turns upwards where *k* is small, resulting in a strip of parameter space where small *k* produces unstable spirals, regardless of *n*. It is at this point that the behaviour does not correspond to the eigenvalues’ prediction.

It is important to observe that if either *n* or *k* is too small, the system is unstable and does not converge. In fact, convergence is only found where *n* > 1. In other words, the magnitude of the loss must not be too small, and the exponent of the loss term must be greater than 1. The latter result is in agreement with our previous findings on the relevance of the loss exponent *n* to plasma stability^42^ and with a value of *n* > 1 reflecting the order of the reaction leading to electron loss.

**Comparison with observations**. The phenomenon we seek to explain is manifested in several ways: the density of the plasma^19^, defined as the number of electrons in unit volume, a quantity equal to the ion charge density per unit volume because of plasma quasi-neutrality; the intensity of scattering of a laser beam directed through the plasma^19^,^22^ and of the absorption of a laser beam^22^,^46^, both of which are dependent on the dust particle size and density; the intensity of spontaneous light emission from the plasma^21^, dependent on both the plasma density and the dust particle size and density; the unprocessed precursor gas partial pressure or concentration^22^ where the rate of consumption is dependent on the concentration of precursor gas molecules; the mass-spectrometer signal of the active gas molecule^21^; and the absorbance at a particular IR frequency^24^ as well as IR absorption spectra that show the chemical signature of dust particles and precursors^20^,^24^. Table 1 shows the relation of the populations to the measured quantities.

Our model predicts trends in dusty plasma behaviour in good agreement with published observational data. The most direct measurements of the electron density *x*_*1*_, via resonance frequencies in a microwave cavity, have been shown to oscillate with time, and the presence or absence of damping in the oscillations has been shown to depend on plasma parameters^19^, consistent with our model’s solutions in the stability behaviour classes of the ‘neutral centre’ and the ‘stable spiral’.

An interesting feature of the solution for the predator population as a function of time is its tendency to approach toward zero during each oscillation, for some combinations of parameters, while the prey population remains significantly above zero. This scenario is consistent with the observation of Cavarroc *et al*. of periodic void (dust-free region) formation at the centre of a dust-forming plasma^26^.

Figure 5a shows laser light intensity scattered and transmitted from an Ar/C_2_H_2_ discharge, while it is producing dust particles. The intensity of the scattered laser light is a measure of *x*_*2*_, the number density and size of dust particles^22^, as set out in Table 1. Therefore, when the scattered signal oscillations die away, we can infer that the oscillations in dust population are also dying away, in good agreement with the model’s ‘stable spiral’ solution, shown in Fig. 4 as well as in Fig. 2b.

Figure 5b shows the luminous intensity *l* of an Ar/C_2_H_2_ discharge (open circles), as well as the mass spectrometer signal of C_2_H_2_ (filled circles). Both signals oscillate over tens of seconds, decreasing in amplitude over time. As set out in Table 1, light emission is proportional to the number of atoms and ions capturing or being excited by electrons and thus to the electron population *x*_*1*_. However, the presence of dust particles in the plasma has the effect of attenuating the optical signals used for characterisation^47^. Therefore, we define the luminous intensity *l* as *w*_*1*_*x*_*1*_ − *w*_*2*_*x*_*2*_, where *w*_*1*_ and *w*_*2*_ are fitting parameters that determine the relative weighting of the electron density *x*_*1*_ and the dust particle mass per unit volume *x*_*2*_.

The mass spectrometer signal arising from C_2_H_2_ derived species indicates the concentration of C_2_H_2_ molecules present in the plasma. These species are precursors to the carbonaceous particulates, or dust, which form in the plasma, as set out in Fig. 1, where C_2_H_2_ molecules are dissociated by collision with electrons and then agglomerate to form more massive particles. Hence the C_2_H_2_ derived signal reflects only the population of the dust particulate *precursors*, and the rate of consumption of C_2_H_2_ is proportional to the electron population in the plasma. Therefore, the C_2_H_2_ signal corresponds to the negative integrated electron population, such that the maxima in electron population align with the fastest decrease in C_2_H_2_ population.

In Fig. 5, the light emission and mass-spectrometer data shown in b can be compared with the calculations shown in c, which shows the corresponding quantities of *l* and the negative integral of the prey population curve, as calculated from equation pair [2]. The simulation is consistent with experiment, as both show damped oscillations that appear to approach steady state, and the calculated phase delay is such that the maxima in electron population align with the fastest decrease in C_2_H_2_ concentration, as seen experimentally.

**Oscillation frequency**. The dust creation rate parameter δ is primarily determined by the precursor gas concentration, or, more specifically, the concentration of precursor elements. δ increases from a CH_4_ plasma to a C_2_H_2_ plasma, because C_2_H_2_ has twice as much carbon per molecule and thus is likely to be more effective at making dust. One event of an electron encountering a carbonaceous particle produces twice as much carbon which can then be used to agglomerate to form larger dust particles.

Figures 5b and d show light emission and mass-spectrometer signals for the active gas in plasmas of two different compositions, Ar/C_2_H_2_ and Ar/CH_4_, measured on the same system. Measurements of the Ar/C_2_H_2_ plasma show much faster oscillation than those for the Ar/CH_4_ plasma, as can be expected from the higher concentration of carbon atoms. A similar result has been reported experimentally, namely, that the period of oscillation of the electron density *x*_*1*_ depends inversely on the fraction of the reactive gas in the gas mixture^19^. (No pressure dependence has been reported, other than a threshold pressure below which oscillations are not seen; above the threshold, the period of oscillation does not vary with pressure^19^).

The calculated periodicity is affected by the dust creation rate parameter δ according to Fig. 6a, where a change in δ whilst holding the other parameters constant results in a change in period of oscillation in a quasi-parabolic shape. The data points shown here are calculated by averaging the first five periods of oscillation for each δ. Where the period decreases with increasing δ, the period of oscillation is relatively constant over the first five calculated periods of oscillation, as is seen experimentally. This left-hand side of the paraboloid can be thought of as the physically realistic side, because it corresponds to what is seen experimentally. The right-hand side of the paraboloid, where the period increases with increasing δ is effectively a mathematical artefact, and can be thought of as non-physical. On this side, for each δ, the period decreases considerably over the first five oscillations, by up to 50% of the duration of the first oscillation.

The period of electron density fluctuation has been shown experimentally to depend inversely on the gas flow rate (Fig. 6c) as well as inversely on the silane fraction^19^, and it has been noted elsewhere that a lower flow rate of SiH_4_ results in slower oscillations^46^. The gas flow rate influences δ, because it is the presence of the active gas that allows the creation of dust, according to the model described in Fig. 1. Hence the experimental curve of Fig. 6c corresponds to the left-hand side of the paraboloid in Fig. 6a.

The electron birth rate parameter α depends on both the rf power supplied to the plasma and the pressure, as these determine the rate at which electrons can create new electrons. The power determines how much energy the electrons are given, which in turn determines the rate at which electrons create new electrons. Pressure determines the mean free path and thus the frequency of collisions of an electron with a given energy. Both of these effects increase the electron concentration *x*_*1*_, which is reflected in an increased rate of growth of the particles. The increased rate of growth increases the frequency of the oscillations. Figure 6b shows the effect of α on the period of oscillation, as calculated from equation pair [2]. For comparison, experimental data show that rf power affects the period of electron density fluctuation as shown in Fig. 6d, where period decreases with increasing rf power until it reaches a minimum and then increases with increasing rf power. The calculated inverse relation of b corresponds to the initial decrease seen experimentally in d.

The predation rate β corresponds to the particles’ affinity for electrons, which would vary for different species of predator. For example, a silicon-based predator such as silane would have a different predation rate of electrons from a carbon-based predator such as methane or acetylene. A particularly interesting case to consider is that of candle flames, where the particles (predators) create electrons (prey) directly, via thermionic emission from the soot. It is a further twist on the classic Lotka-Volterra relation, a complement to the situation set out by Koren and Feingold in the context of aerosol-cloud-precipitation systems, where the prey (cloud) spawns the predator (rain), as raindrops coalesce from cloud droplets^41^. In a candle flame where the electron emission is stronger than the electron predation because of thermionic emission, then β would have a negative sign, indicating creating rather than destroying. In this case, it is still possible to attain stability, as *n* and *k* determine the death rate of the electrons, rather than β. In fact, the eigenvalues as set out in equation [6] happen not to include β, so its sign or indeed even its magnitude does not appear to affect the stability behaviour at all.

The essence of the predator-prey problem, namely, that a population does something in their own interest, short-term, that has the longer-term effect of being detrimental to them, is a phenomenon that appears in a wide variety of fields. It is manifested in this work by the formation of predator-free regions (voids) in dusty plasmas. We have demonstrated a natural way of inducing stability in the predator-prey problem by nonlinear losses, which we commend for wider application. The phenomenon of the electron (prey) population reaching its maximum just before the particle (predator) population is elegantly captured by the predator-prey model in a way no other model does. The success of ecological population dynamics in describing the population interactions of dust-forming plasmas may serve as an inspiration for others who seek insight via interdisciplinary integration.

## Additional Information

**How to cite this article**: Ross, A. E. and McKenzie, D. R. Predator-prey dynamics stabilised by nonlinearity explain oscillations in dust-forming plasmas. *Sci. Rep.* **6**, 24040; doi: 10.1038/srep24040 (2016).

## Figures and Tables

**Figure 1:**
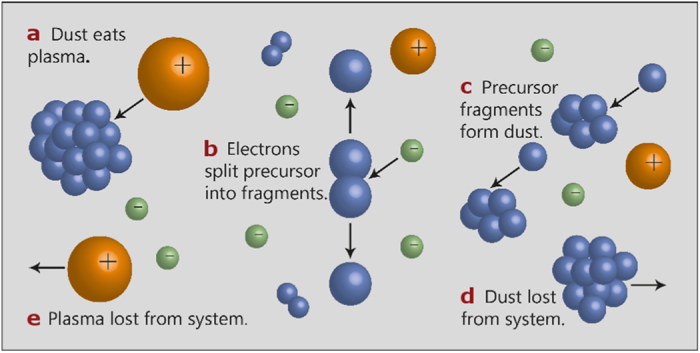
An overview of the processes occurring in a dusty plasma. (**a**) Dust particles effectively ‘eat’ or consume plasma as a cumulative effect of the following processes that occur in a dusty plasma: (**b**) Electrons collide with particles of the precursor gas, such as silane, methane, or acetylene, splitting them into reactive fragments. (**c**) The resulting fragments coalesce to form dust particles. (**d**) Once a dust particle grows too large, it is lost from the system. (**e**) Plasma is lost from the system, through recombination of ions and electrons or loss to the environment of the plasma. Visualisation based on Hollenstein^12^.

**Figure 2:**
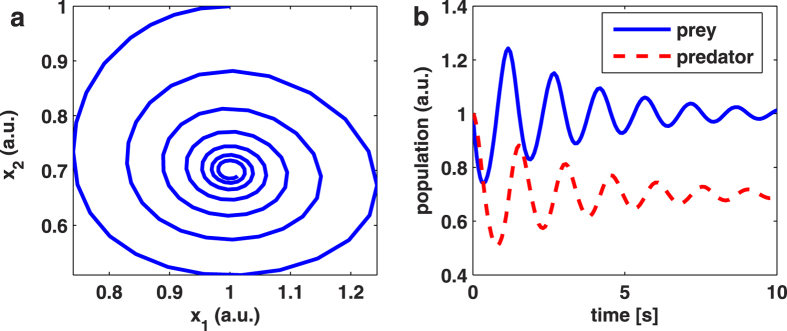
Example solution to the modified Lotka-Volterra equations, showing simulated behaviour of a predator-prey relationship applicable to dusty plasma populations. Parameters used were: α = β = γ = δ = 5; *k* = 1.5; *n* = 1.4; and the starting point for calculation was (*x*_*1*_, *x*_*2*_) = (1, 1). (**a**) The predator (*x*_*2*_) and prey (*x*_*1*_) populations begin at (1, 1) and spiral in to a stable value. (**b**) Time series of predator and prey, where the solid blue line indicates the prey population and the dashed red line indicates the predator population. The populations oscillate out of phase, with damping, before stabilising to an equilibrium.

**Figure 3:**
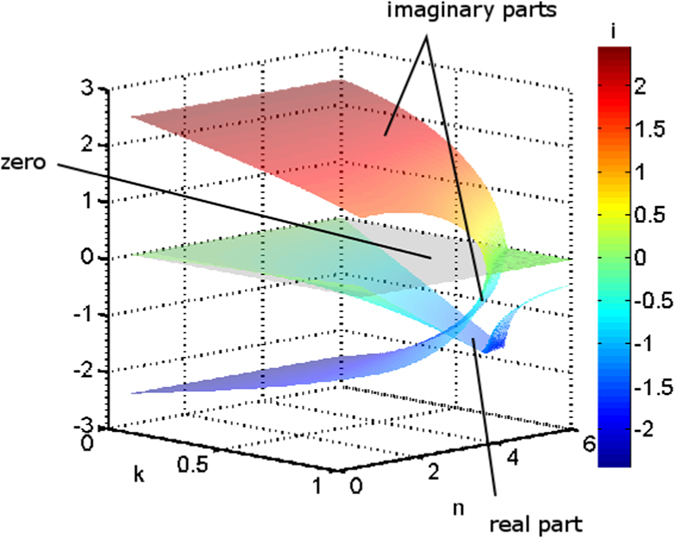
Eigenvalues of the Jacobian (eq. 5) as a function of *n* and *k*, as given by equation 6, with real and imaginary parts plotted on the same *z* axis. The data shown here were calculated with α = β = 1.5, and γ = δ = 4; the eigenvalue plot is different for each combination of α, β, γ, and δ. The imaginary parts of each pair of eigenvalues mirror each other across the *n-k* plane, which is indicated where the *z* axis is zero. The real part of the eigenvalues reaches a minimum where the imaginary parts reach zero, where *n* and *k* are both large. Beyond this line, the imaginary parts remain zero, and the real part remains negative but approaches zero.

**Figure 4:**
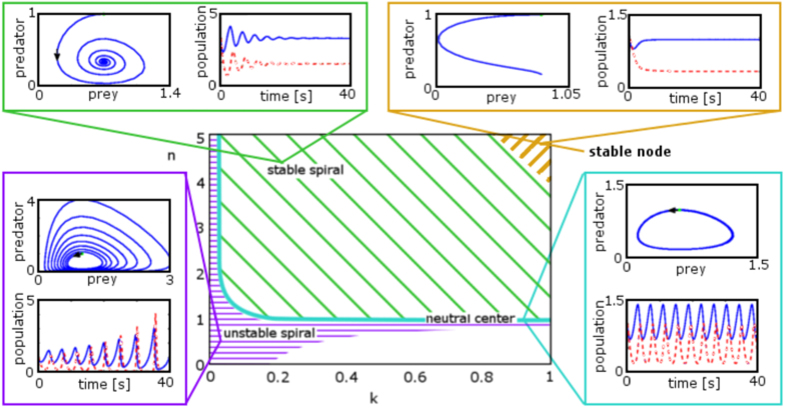
The range of behaviours of solutions to equation pair [2] is shown here as functions of *n* and *k* for solutions where α = β = 1.5, and γ = δ = 4. The behaviour plot is different for each combination of α, β, γ, and δ. This set of parameters allows a wide range of behaviours to be observed in a relatively small *n*-*k* parameter space. The stability of the system falls into four categories, based on the predator-prey population, or limit-cycle, graph behaviour. An ‘unstable spiral’ spirals away from the initial condition, yielding population oscillations that grow without bound. A ‘neutral centre’ indicates a closed loop, where population oscillations stay steady without damping. A ‘stable spiral’ spirals in to reach a point of stability, and population oscillations are damped until both populations remain steady. A ‘stable node’ indicates movement towards a stable point without oscillation, with both populations equilibrating quickly.

**Figure 5:**
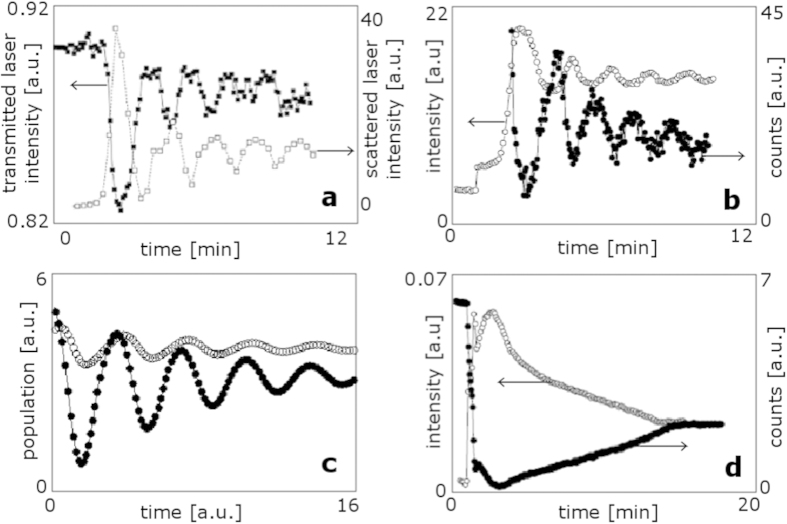
Oscillations in dust-forming plasmas. (**a**) Laser light intensity transmitted through (■) and scattered from (□) an Ar/C_2_H_2_ discharge. From Hong *et al*.^21^, reproduced with permission. (**b**) Measurements of light emission (○) from an Ar/C_2_H_2_ discharge and the mass spectrometer signal of C_2_H_2_ (●) in the discharge^21^. Reproduced with permission. (**c**) The quantities calculated from equation pair 2 corresponding to the measured quantities in (**b**): light emission *l* (○) and the negative integral of the light curve (●), corresponding to the active gas or precursor population, whose rate of consumption is proportional to the electron (prey) population. (**d**) Light emission measurements (○) from an Ar/CH_4_ discharge and the mass spectrometry measurements of CH_4_ in the discharge. From Hong *et al*.^21^, reproduced with permission. Note the lower oscillation frequency with respect to the Ar/C_2_H_2_ discharge of part (**b**).

**Figure 6:**
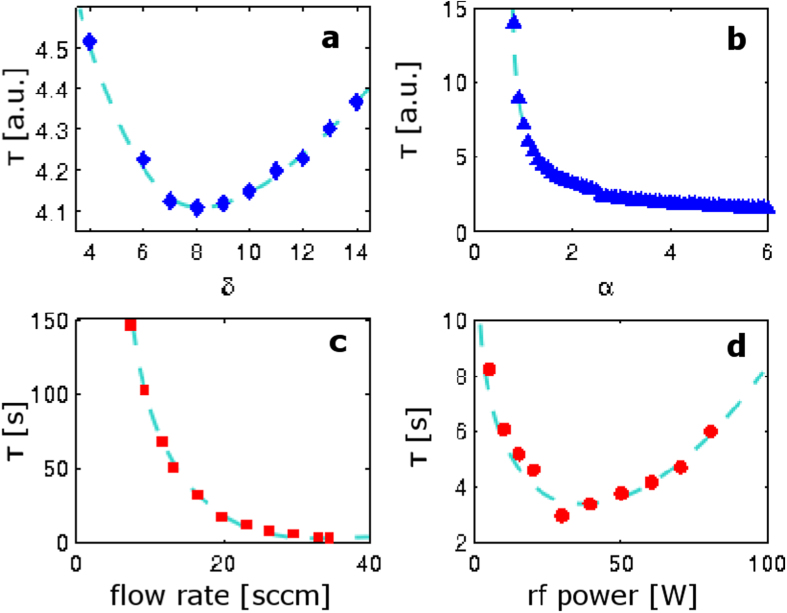
Comparison between experiment and simulation. (**a**) A change in the dust creation rate parameter δ changes the period of oscillation of the solution. Data points are calculated by averaging the first five periods of oscillation at each δ value. Calculations were performed with *k* = 1, *n* = 1.4, α = β = 1.5, and γ = 3. (**b**) The period of oscillation is inversely proportional to the electron birth rate parameter α. Calculations were performed with *k* = 1, *n* = 1.4, β = 1.5, γ = 3, and δ = 8. (**c**) Experimental data showing that the gas flow rate in standard cubic centimetres per minute (sccm) has an inverse relationship to the oscillation period. Data from Stoffels *et al*.^19^, used with permission. (**d**) Experimental data showing the inverse relation between the period of oscillation and the rf power. Data from Stoffels *et al*.^19^, used with permission. Trendlines are drawn to aid the eye.

**Table 1:** Model parameters and measureable quantities.

Term	Quantity	Method of evaluation
x_1_	Electron density (equivalent to ion density)	Resonance frequency of the chamber used as a microwave cavity
x_2_	Dust particle mass per unit volume	Transmitted and scattered light intensity
α	Electron birth rate parameter	Primarily influenced by power input to the plasma and confining magnetic field
β	Predation rate (rate at which dust particles consume plasma)	Primarily influenced by chemical makeup of dust and its affinity for electrons
γ	Dust loss rate parameter	Influenced by plasma geometry, especially size and boundary conditions
δ	Dust creation rate	Determined by precursor gas concentrations (input flow rate)
*k*	Electron and ion loss parameter	Influenced by plasma geometry, especially any confining magnetic field
*n*	Electron and ion loss exponent	Model parameter reflecting the order of the reactions that cause loss
*l*	Luminous intensity of the plasma	Determined by the weighted difference between x_1_ and x_2_
*c*	Precursor gas concentration	Primarily determined by precursor gas flow rate
